# Malaria and hypertension. Another co-evolutionary adaptation?

**DOI:** 10.3389/fcimb.2014.00121

**Published:** 2014-09-03

**Authors:** Julio Gallego-Delgado, Ana Rodriguez

**Affiliations:** Division of Parasitology, Department of Microbiology, New York University School of MedicineNew York, NY, USA

**Keywords:** malaria, plasmodium, hypertension, angiotensin II, polymorphism, ethnicity, evolutionary adaptation

Arterial hypertension is a complex multifactorial disease and a global public health concern. It is responsible for at least 45% of deaths due to heart disease and 51% of deaths due to stroke, adding up the tremendous number of 9.4 million deaths every year (Lim et al., 2012; World Health Organization, 2013). The renin-angiotensin-aldosterone system (RAAS) is one of the most important regulatory systems of blood volume, arterial pressure and cardiovascular homeostasis. Angiotensin II (Ang II) is the principal effector hormone of the RAAS in vascular biology, mediating effects via two main receptors: Angiotensin receptor type 1 (AT1) and type 2 (AT2) (Callera et al., 2007). When Ang II binds to AT1 on vascular smooth muscle cells, it mobilizes intracellular Ca^2+^, leading to cellular contraction. Sustained cellular contraction increases peripheral vascular resistance, resulting in high blood pressure (Touyz and Schiffrin, 2000).

Among others, genetic factors are closely related with the development of hypertension. People with African American and South Asian genetic background have higher prevalence of hypertension compared to Caucasians, independently of their socioeconomic status (Cappuccio, 1997; Sampson et al., 2014). These differences in the ethnic background can increase the prevalence of hypertension by 2 fold until the age of 55, when the differences decline, presumably because of the interfering effect of age-related factors (Wolz et al., 2000). Several polymorphisms have been associated with higher prevalence of arterial hypertension; there are 2 polymorphisms in the Angiotensin Converting Enzyme (ACE) and Angiotensin Converting Enzyme 2 (ACE2) that lead to elevate circulating Ang II levels (Giner et al., 2000; Di Pasquale et al., 2004; Fan et al., 2007). Interestingly, these same genetic variations (the “D” allele of ACE I/D polymorphism and the ACE2 C→T substitution) have been associated with a lower incidence of cerebral malaria (CM) in Indian adults, although the later one only in women (Dhangadamajhi et al., 2010).

Malaria is still a major public health problem world wide, causing approximately 600,000 deaths, mostly among African children (World Health Organization, 2012a). A high proportion of these deaths are caused by CM, a syndrome characterized by impaired consciousness, generalized convulsions, coma and neurological sequelae (Idro et al., 2005). CM is caused by the interaction between *Plasmodium falciparum* infected erythrocytes and host brain endothelial cells. Parasite proteins that are expressed on the surface of infected erythrocytes (PfEMP1), interact with host endothelial cell receptors (Protein C receptor, ICAM-1; Newbold et al., 1997; Turner et al., 2013) leading to their sequestration within the brain microcirculation. Disruption of the blood-brain barrier is observed producing the characteristic petechiae and ring hemorrhages found on the brain of dead patients with CM (Rasti et al., 2004).

Although it is still not well-established that Ang II has beneficial effects on malaria and particularly on CM, different lines of evidence suggest a possible ‘protective’ effect that could be mediated by different, non-exclusive mechanisms that could affect parasite development and/or host susceptibility to *Plasmodium-induced* pathology.

Recent reports have shown that angiotensin peptides can induce impairment of the erythrocytic cycle of *Plasmodium*, reducing the parasite growth *in vitro* (Maciel et al., 2008; Saraiva et al., 2011). Since the development of CM depends on the initial levels of parasitemia in mice (Amani et al., 1998) and possibly in humans (Bejon et al., 2005), this could be a potential explanation for Ang II protection from CM. Our unpublished results using mice infected with *Plasmodium berghei* seem to confirm that the elevation of Ang II levels results in modestly decreased parasitemias in this animal model.

It is possible that Ang II could modulate malaria severity through additional mechanisms, specially, since the inhibitory effect observed in parasite growth is modest (Maciel et al., 2008; Saraiva et al., 2011). Sodium conservation could provide an alternative explanation to the protective effect of the polymorphisms associated with higher levels of Ang II. In this case, Ang II could be acting to counterbalance hyponatremia by stimulating the secretion of aldosterone from the adrenal cortex, the major regulator of Na^+^ reabsorption. Hyponatremia (serum sodium < 135 mmol/L) is frequent in malaria patients and correlates with disease severity in imported malaria (van Wolfswinkel et al., 2010). However, other studies have shown that circulating Na^+^ levels have no predictor value for the outcome of malaria (Phillips et al., 2009) and are not associated with increased mortality (Hanson et al., 2009). Further investigations are necessary to elucidate the role of serum Na^+^ levels in the outcome of severe malaria and its possible relation with polymorphisms of the RAAS.

A protective effect of Ang II against CM may also be mediated by effects on the host vascular endothelial cells. Ang II exerts its vasoconstriction effect through the AT1 receptor on vascular smooth muscle cells (de Gasparo et al., 1990). However, since the BBB is impermeable to Ang II and other RAAS peptides, circulating Ang II does not reach underlying vascular muscular cells and vascular pressure in the brain is locally regulated by Ang II produced within the brain parenchyma (Bader, 2010). In the context of CM, higher levels of circulating Ang II would not increase vasoconstriction in the brain, but could affect endothelial cells that form the BBB and express both, AT1 and AT2 receptors. Using monolayers of human brain microvascular endothelial cells, we have observed that incubation with erythrocytes infected with *Plasmodium falciparum* promotes the disruption of interendothelial cell junctions between these cells. Activation of AT2 or inhibition of AT1 receptor preserves the integrity of interendothelial cell junctions after incubation with *P. falciparum in vitro* and protect against experimental CM in mice (Gallego-Delgado et al., 2013). This could be key in the association of Ang II with lower development of CM since AT2 receptor stimulation has protective effects on brain injury (McCarthy et al., 2009; Habashi et al., 2011) and inhibits vascular endothelial cells migration (Falcon et al., 2005). Gene expression analysis comparing CM-susceptible mice vs. CM-resistant mice also showed higher expression levels of the AT2 receptor gene in CM-resistant mice compared to susceptible ones (Delahaye et al., 2007). Also, activation of AT2 receptor results in increased production of nitric oxide by endothelial Nitric Oxide Synthase (eNOS) in endothelial cells (Yayama and Okamoto, 2008), which could be protective against cerebral malaria since low nitric oxide bioavailability may exacerbate endothelial dysfunction and contributes to the pathogenesis of severe malaria (Gramaglia et al., 2006; Miller et al., 2013). Interestingly, polymorphisms in eNOS that are responsible for increased expression and nitric oxide production have been associated with mild malaria (Dhangadamajhi et al., 2009, 2010).

Finally, it has also been proposed that the effects of Ang II in malaria severity could be mediated by immune mechanisms, since activation of AT1 by Ang II is a pro-inflammatory stimulus (Benigni et al., 2010). In this case, elevated levels of Ang II would result in higher inflammation that would contribute to the activation of endothelial cells and would be detrimental for the outcome of severe malaria. Treatment of *Plasmodium*-infected mice with losartan, an inhibitor of AT1, and captopril, an inhibitor of ACE that reduces the levels of Ang II, inhibit T cell activation induced by infection (Silva-Filho et al., 2013). Treatment with losartan, which inhibits the signaling of Ang II through AT1, while potentiating the effects mediated by AT2, would result in decreased endothelial cell migration and lower disruption of interendothelial cell junctions, in addition to anti-inflammatory systemic effects (Marchesi et al., 2008) and would align with the hypothesis of a beneficial effect of Ang II in malaria severity. However, the effects of captopril, reducing Ang II, decreasing parasitemia and protecting against experimental CM (Silva-Filho et al., 2013), apparently do not agree with the hypothesis that increased Ang II provides protection against severe malaria. Further experiments *in vivo* are necessary to elucidate the signaling pathways, AT1 vs. AT2, mediating the observed effects on malaria severity of different concentrations of systemic Ang II in parasite development and generation of CM.

Humans have been infected by *P. falciparum* for over 50,000 years (the estimated date for the out-of-Africa migration) and have co-evolved during this time (Tanabe et al., 2010). Different human polymorphisms, such as those causing thalassemia or sickle cell anemia, have been associated to this natural selection process. Although there is not sufficient evidence available to demonstrate it, the association of polymorphisms of the ACE and protection from severe malaria may also be the result of a natural selection process. This apparent contradiction would be explained if higher levels of Ang II at early ages protect from CM. In this case, those polymorphisms would have a higher transmission ratio, despite their deleterious effects during adulthood, as observed in people with African American genetic background (Sampson et al., 2014).

While hypertension has been traditionally considered a disease of western countries, in the latest years it has become obvious that hypertension is coming to epidemic levels in Africa, even presenting higher prevalence than in wealthier countries, possibly because of the higher levels of circulating Ang II found in people with African genetic background (Addo et al., 2007; Lloyd-Sherlock et al., 2014). As a consequence, a significant number of African natives are currently taking anti-hypertensive medication, and this number is expected to increase exponentially in the coming years (Lloyd-Sherlock et al., 2014). If further investigations demonstrate that Ang II levels influence the development of CM, hypertension treatment in malaria endemic areas should take this effect into consideration.

Finding polymorphisms associated to hypertension in Sub-Saharan Africa could help to select the adequate antihypertensive treatment in these highly vulnerable populations (Mokwe et al., 2004; Peck et al., 2013). The fact that differences in the ethnic background correlate with differences in the response to antihypertensive drugs suggests that the genetic background could be an essential factor to consider when it comes to choose the better antihypertensive regimen to control blood pressure (Gupta, 2010; Gupta et al., 2010).

Another population possibly affected by the influence of anti-hypertensive medication on malaria severity would be travelers from western countries going to malaria endemic areas. Nonimmune malaria patients, such as travelers, are more susceptible to succumb to CM and develop neurological sequelae (Alrajhi et al., 1999; Roze et al., 2001). But not all nonimmune travelers have the same odds to develop malaria complications. In a 16-year period clinical study of natives from malaria-nonendemic countries infected with malaria, Phillips et al. showed that the odds of severe malaria were 8-fold lower among black than other ethnic groups suggesting genetic factors to contribute to the outcome of the disease (Phillips et al., 2009). WHO estimates that over 100 countries with risk of malaria transmission receive 125 million travelers every year and reports 10,000 cases of malaria among them (World Health Organization, 2012b). Given that ~20% of the adult population in western countries is hypertensive, a significant number of those travelers would be taking anti-hypertensive medication while in malaria endemic territories. A first line therapy for hypertension treatment are the Angiotensin converting enzyme inhibitors (ACEi) that inhibit the production of Ang II from angiotensin I. However, in malaria endemic areas, reducing the levels of systemic Ang II could be reducing the chances of a good outcome in the case of a malaria infection. Alternative anti-hypertensive drugs, that do not target ACE, for hypertensive travelers would be decreasing the chances of developing CM in case of acquiring malaria.

Overall, there are evidences suggesting that RAAS, and more specifically Ang II, may influence the severity of malaria. Although the available data are still very limited, it is conceivable that Ang II could be protecting the integrity of the BBB in the setting of CM and giving a survival advantage to those that carry polymorphisms resulting in higher levels of systemic Ang II. Mechanistic studies to determine the role of Ang II in inflammation, endothelial cell barrier formation, sodium conservation and parasite growth in the context of malaria are needed before any solid conclusions can be reached. It is possible that polymorphisms acquired because of their protective effect against malaria have turned out to contribute together with the western societies life-style to the worldwide epidemic proportions of hypertension. However, the available data in this sense are still scarce and additional genetic studies are necessary in other malaria endemic regions (specially in sub-Saharan Africa) to determine whether malaria could have been a driving evolutionary force for RAAS polymorphisms, and therefore for hypertension.

## Conflict of interest statement

The Guest Associate Editor Samuel C. Wassmer declares that, despite being affiliated to the same institution as the authors, the review process was handled objectively and no conflict of interest exists. The authors declare that the research was conducted in the absence of any commercial or financial relationships that could be construed as a potential conflict of interest.
